# Rapid Detection of the Anti-Tumor Drug Etoposide in Biological Samples by Using a Nanoporous-Gold-Based Electrochemical Sensor

**DOI:** 10.3390/molecules29051060

**Published:** 2024-02-28

**Authors:** Huiyuan Yu, Mengjie Hu, Xiaolei Wang, Xia Wang, Luying Xun, Honglei Liu

**Affiliations:** 1 State Key Laboratory of Microbial Technology, Shandong University, 72 Binhai Road, Qingdao 266237, China; yhyuan@mail.sdu.edu.cn (H.Y.); humengjie@sdu.edu.cn (M.H.); 202212540@mail.sdu.edu.cn (X.W.); ghwx@sdu.edu.cn (X.W.); luying_xun@vetmed.wsu.edu (L.X.); 2 School of Molecular Biosciences, Washington State University, Pullman, WA 99164-7520, USA

**Keywords:** electrochemical sensor, nanoporous gold, etoposide, anti-tumor

## Abstract

Monitoring etoposide is important due to its wide usage in anti-tumor therapy; however, the commonly used HPLC method is expensive and often requires complicated extraction and detection procedures. Electrochemical analysis has great application prospects because of its rapid response and high specificity, sensitivity, and efficiency with low cost and high convenience. In this study, we constructed a nanoporous gold (NPG)-modified GCE for the detection of etoposide. The electrochemical oxidation of etoposide by NPG caused a sensitive current peak at +0.27 V with good reproductivity in 50 mM of phosphate buffer (pH 7.4). The relationship between etoposide concentration and peak current was linear in the range between 0.1 and 20 μM and between 20 and 150 μM, with a detection sensitivity of 681.8 μA mM^−1^ cm^−2^ and 197.2 μA mM^−1^ cm^−2^, respectively, and a limit of detection (LOD) reaching 20 nM. The electrode had a good anti-interference ability to several common anions and cations. Spiked recovery tests in serum, urine, and fermentation broth verified the excellent performance of the sensor in terms of sensitivity, reproducibility, and specificity. This may provide a promising tool for the detection of etoposide in biological samples.

## 1. Introduction

Etoposide (ETO), also known as V-16, is an anti-tumor drug approved by the US Food and Drug Administration in the treatment of refractory testicular tumors, small-cell lung cancer, and several other tumors and in chemotherapy for autologous stem cell transplantation [1]. It is obtained from a derivation of podophyllotoxin, an aryl-tetralin-type lignan extracted from the roots and rhizomes of Mayapple plants [2]. As a DNA topoisomerase Ⅱ inhibitor, etoposide inhibits the ability of topoisomerase Ⅱ to change the topological conformation of DNA, causes the fragmentation of DNA, and therefore blocks the cell cycle, leading to the death of cancer cells [3]. Recently, scientists found that the drug may have other pharmacological effects. It is used to treat immune-mediated inflammatory diseases associated with cytokine storm syndrome [4]. In the COVID-19 epidemic, etoposide was proven to dramatically reduce symptoms in patients presenting with complications of recurrent cytokine storms [5,6]. Although etoposide has a lot of benefits, its long-term use is associated with secondary leukemia, and the reactive oxidative metabolites of etoposide could alter the proliferation and differentiation of hematopoietic stem cells, which makes the drug a double-edged sword [7]. Due to its wide usage and double effects in clinics, the monitoring of the presence of etoposide in the production process and its metabolism in the human body is of great importance.

Many methods can be used in etoposide detection. Among them, high-performance liquid chromatography (HPLC) and liquid chromatography–mass spectrometry (LC-MS) are commonly used in production and research processes. For example, Renata et al. extracted etoposide from dried blood and then detected its content in cancer patients using the HPLC method [8]. Usually, the detection of etoposide via HPLC needs liquid–liquid extraction with an organic solvent, which is trivial and troublesome [9]. Other, new methods were also applied in etoposide detection. Soetebeer et al. used capillary electrophoresis with UV-laser-induced native fluorescence to detect etoposide and etoposide phosphate in human plasma [10]. Ragozina et al. employed micellar electrokinetic chromatography in combination with near-field thermal lens detection (NF-TLD) for the rapid simultaneous determination of etoposide phosphate and etoposide in human blood plasma [11]. The surface plasmon resonance method was also applied to analyze the content of etoposide in urine [12]. However, these methods are often complicated and expensive in both sample preparation and operation, requiring expensive instruments and professional operators, which are not suitable for the fast on-site detection of etoposide in biological samples.

The use of electrochemical sensors to monitor the concentrations of molecules of biological relevance has been the object of increasing investigation in electroanalytical research. The electrochemical method shows the great advantages of rapid response and high specificity, sensitivity, and efficiency, with low cost, portable convenience, and no professional operator requirements [13]. Several electrochemical sensors are already available for anti-tumor drug determination, with the typical modification of electrodes [14]. Zhu et al. developed an electrochemical sensor from the modification of a glass carbon electrode (GCE) with multiwalled carbon nanotubes, functionalized with quaternary amine (q-MWNTs), to determine the content of methotrexate (NTX) and folic acid [15]. Zhang et al. synthesized a graphene nanoribbon nanocatalyst (GNR) and modified a glassy carbon electrode (GCE) with GNR to detect etoposide in biological fluids using the differential pulse voltammetry (DPV) method [16]. A mercury-film-coated graphite pencil electrode (MF/GPE) was prepared by Temerk et al. and applied for selective and sensitive electrochemical determination of the anti-cancer drug lomustine (LMT) [17]. Radhapyari et al. developed an amperometry biosensor based on a polyaniline–gold nanocomposite film coated with horseradish peroxidase for the detection of the anti-cancer drug gemcitabine in bulk and its parenteral formulation [18]. Some electrochemical sensors were also developed for the determination of etoposide [19]. These applications have proved that the modification of materials and structures of the electrodes can effectively improve the sensor’s performance.

Metal nanoparticles have been widely used in electrochemical analysis due to their catalytic properties and high specific surface [20,21]. Nanoporous gold (NPG), a three-dimensional nanomaterial with tunable porosity and excellent electrochemical properties, has been widely applied in constructing sensors [22]. The commonly used method to prepare NPG is the dealloying method, which selectively dissolves the more reactive component of the metal alloy materials, with gold remaining in the porous structure [23]. NPG has the characteristics of high specific surface area, good electrical conductivity, chemical stability, and biocompatibility. Therefore, it is often used to modify the electrodes [24].

In this work, we constructed an electrochemical sensor NPG/GCE using glassy carbon electrodes (GCEs) modified by NPG for the detection of etoposide. The morphology of the electrodes was examined using the electron microscopic method; its electrochemical behavior, anti-interference ability, and reproducibility were characterized using cyclic voltammetry (CV) and differential pulse voltammetry (DPV) methods; and the practical performance of the electrodes was verified in biological samples.

## 2. Results and Discussion

### 2.1. Construction and Characterization of NPG/GCE

**Figure 1:**
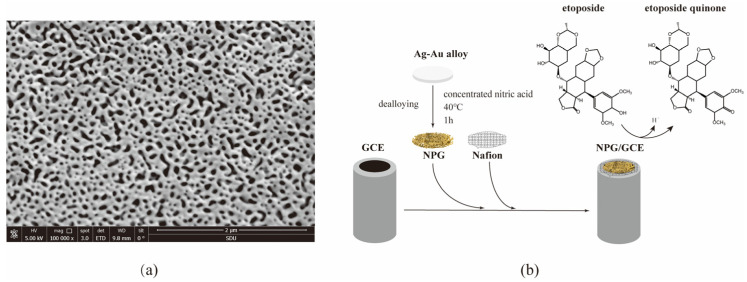
The construction of the NPG/GCE. (**a**) The SEM image of the NPG film; (**b**) the construction of the NPG/GCE.

NPG film was prepared by dealloying Au/Ag film and its morphology was investigated using a scanning electron microscope (SEM). As shown in Figure 1a, the NPG film showed an open three-dimensional gold ligament structure with nanopores of about 30–50 nm on average, which enabled the film to exhibit a large specific area for substrate binding, excellent electrical conductivity, and good electrocatalytic activity with high accuracy and reproducibility [25]. The ligament structure possesses a high density of geometrically required surface steps and kinks that are active for chemical reactions, a high thermal stability against coarsening, and tunable chemical compositions for enhanced catalytic performances [26]. The NPG film was stabilized on the GCE with 0.1% Nafion (Figure 1b). Nafion itself does not interfere with the electrochemical reaction and may help to reduce catalyst NPG detachment from the surface [27].

### 2.2. Characterization of NPG/GCE and the Feasibility of Etoposide Detection Using NPG/GCE

**Figure 2:**
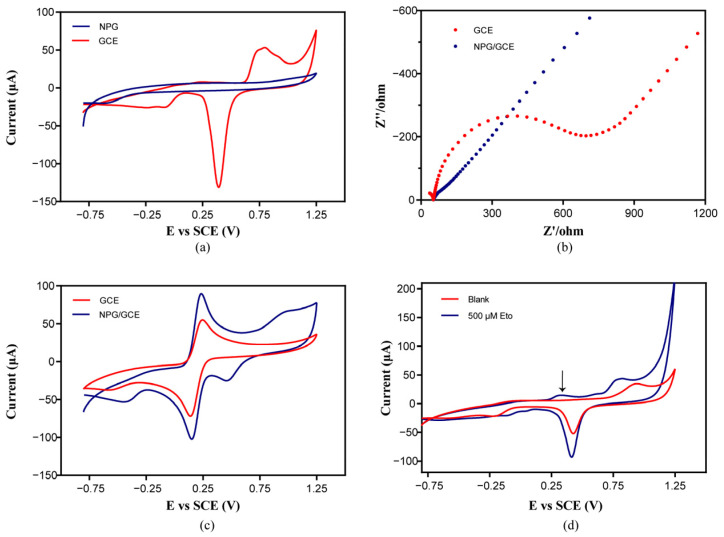
(**a**) The CV curves of the bare GCE and NPG/GCE in phosphate buffer (50 mM, pH 7.4); (**b**) the EIS profiles of the bare GCE and NPG/GCE; (**c**) the CV curves of the bare GCE and NPG/GCE in phosphate buffer (50 mM, pH 7.4) containing 5 mM of potassium ferricyanide and 5 mM of potassium hexacyanoferrate (II); (**d**) the CV curves of the NPG/GCE in phosphate buffer (50 mM, pH 7.4) with and without 500 μM of etoposide at a scan rate of 50 mV s^−1^; the arrow shows the current peak of etoposide.

To investigate the electrochemical performance of the electrodes, the CV response and electrochemical impedance spectroscopy (EIS) of the bare GCE and constructed NPG/GCE were characterized in a three-electrode cell. As shown in Figure 2a, in the CV measurement in 50 mM of potassium phosphate buffer (pH 7.4) at a scan rate of 50 mV s^−1^ from −0.8 V to +1.25 V, an obvious oxidation peak appeared at +0.85 V and a sharp reduction peak appeared at +0.4 V, while the bare GCE showed no apparent peaks. This indicated that the NPG was successfully loaded on the GCE surface with good electrochemical activities [28].

The EIS and CV measurement was further performed in 50 mM of phosphate buffer (pH 7.4) containing 5 mM of potassium ferricyanide and 5 mM of potassium hexacyanoferrate (II). The EIS of the GCE showed a semi-circular curve, while the radius of the semicircle of the NPG/GCE was significantly reduced (Figure 2b), indicating that the conductivity of the NPG/GCE was greatly improved [29]. The oxidation peak of [Fe(CN)_6_]^4−^ and the reduction peak of [Fe(CN)_6_]^3−^ were higher in the CV curve of the NPG/GCE than that of the GCE (Figure 2c), which is consistent with the fact that gold nanoparticles increase the specific surface area, which contributes to good electron transfers [30]. These results proved that NPG modification of the GCE could reduce the impedance, promote electronic transmission, enhance the current signal, and improve the electrochemical catalytic performance of the electrode.

The electrochemical response of the NPG/GCE to etoposide was investigated in 50 mM of potassium phosphate buffer (pH 7.4) using the CV method. As shown in Figure 2d, compared to blank phosphate buffer, the CV curve of 500 μM of etoposide in the same buffer showed an obvious oxidation peak at +0.3 V, indicating that the redox reaction of gold atoms on the surface of the NPG/GCE produced a visible current signal in the CV spectrum. The peak in the CV curve of the GCE was not obvious, indicating that the reaction was catalyzed by the nanoporous gold. The clear signal response provided the possibility of electrochemical detection of etoposide by using NPG/GCEs.

### 2.3. Effect of Electrolyte pH on Etoposide Oxidation

**Figure 3:**
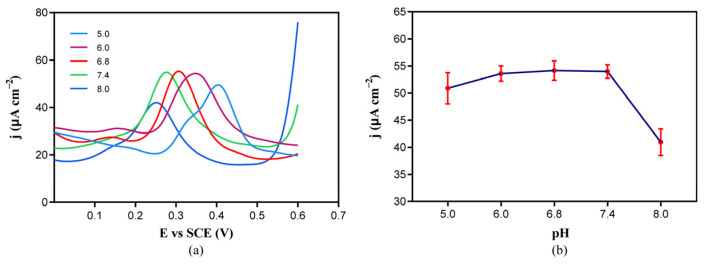
(**a**) The DPV curves of 100 μM of etoposide in 50 mM of phosphate buffer with different pH values (5.0–8.0); (**b**) the change in etoposide oxidation peak current with the change in pH.

To achieve the highly sensitive detection of etoposide in biological samples, we analyzed the effect of electrolyte pH on the current signal in the DPV spectrum of etoposide oxidation to find optimal conditions. The DPV analysis was performed at a scan rate of 50 mV s^−1^ from −0.4 V to +1.0 V. The pH range was selected according to the pH of the biological sample urine (pH 5.0–8.0) and serum (pH 7.35–7.45). We chose 5.0, 6.0, 6.8, 7.4, and 8.0 as the test pH of the electrolyte. As shown in Figure 3, the etoposide oxidation peak moved leftward as the pH value increased, and the current density decreased while pH values were excessively acidic (pH 5) or slightly alkaline (pH 8.0), whereas it was similar under neutral and slightly acidic conditions (pH 6.0, 6.8, and 7.4). The occurrence of the electrochemical reaction is related to the transfer of protons. According to our deduced reaction mechanism, the detection of etoposide proceeds through the dehydrogenation of the hydroxyl group on the benzene ring to produce a free proton, and this dehydrogenation reaction is more likely to occur under alkaline conditions, so the potential difference for the occurrence of the reaction decreases, and E_C_ shifts negatively. This is consistent with our observations. Considering the higher sensitivity at physiological pH 7.4, in this study, we selected 50 mM of phosphate buffer (pH 7.4) for the detection of all the samples.

Reproducibility is an important indicator for evaluating the performance of a sensor. In the above measurement with 50 mM of phosphate buffer (pH 7.4) containing 100 μM of etoposide, the current difference was not significant for the five detections, with a maximum peak current density of 61.83 μA cm^−2^, a minimum of 57.32 μA cm^−2^, and a standard deviation of 3.22% (*n* = 5), which indicated that the reproducibility of the sensor fulfilled the desired requirements (Figure S1).

### 2.4. Electrochemical Detection of Etoposide by NPG/GCE

**Figure 4:**
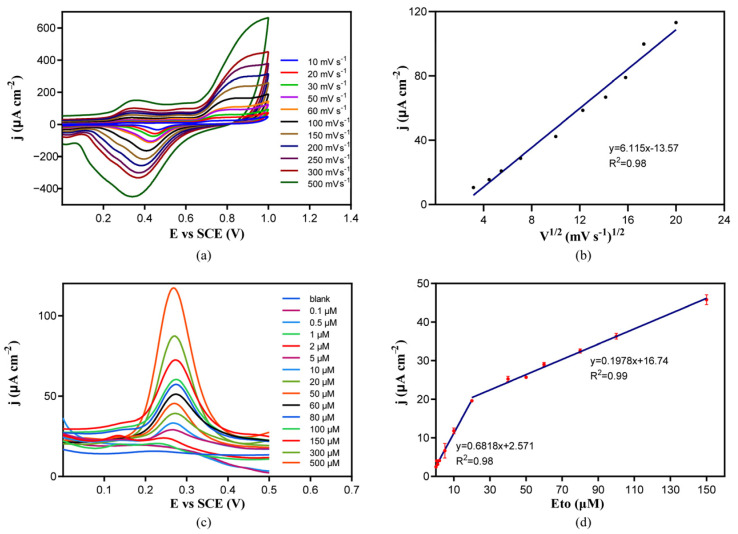
(**a**) The CV curves of the NPG/GCE in phosphate buffer (50 mM, pH 7.4) containing 50 μM of etoposide at different scan rates. (**b**) The linear relationship between the oxidation peak current density of etoposide and the square root of the scan rate for the electrooxidation of etoposide. (**c**) The DPV curves of the NPG/GCE in phosphate buffer (50 mM, pH 7.4) containing different concentrations (0.1 to 500 μM) of etoposide. (**d**) The linear relationship between the oxidation peak current density of etoposide and etoposide concentration, red dots show the average of three replicate measurements and the blue lines indicate the trend lines in two concentration ranges.

To investigate the electrochemical oxidation mechanism of etoposide on the NPG/GCE, the CV curves of 50 μM of etoposide in phosphate buffer (50 mM, pH 7.4) were recorded at different scan rates ranging from 10 mV s^−1^ to 200 mV s^−1^. There is a linear dependence between the etoposide oxidation peak current and the square of the scan rates. As the scan rate increased, the oxidation peak current increased gradually (Figure 4a,b), represented by the following linear regression equation: j (μA cm^−2^) = 6.115 V^1/2^ (mV^1/2^ s^−1/2^)−13.57, (R^2^ = 0.98). The linearity of the dependency may indicate that the etoposide oxidation catalyzed by NPG was a diffusion-controlled process [31]. We hypothesized that NPG catalyzed the oxidation of the phenolic hydroxyl group on etoposide to produce etoposide quinone (Figure 1) in the detection of many phenolic compounds using electrochemical sensors based on the oxidation of the phenolic hydroxyl groups [32,33].

The performance of the NPG/GCE was tested using the DPV method to establish a mathematical relationship between peak current and etoposide concentration. Etoposide was dissolved in dimethyl sulfoxide (DMSO) to prepare a 100 mM stock solution, and it was diluted in 50 mM of phosphate buffer (pH 7.4) to make standard solutions with different etoposide concentrations. Pure DMSO was added to make samples containing the same amount of organic solvent to avoid possible disturbances of DMSO. After DPV analysis at a scan rate of 50 mV s^−1^ from −0.4 V to +1.0 V, the results (Figure 4c) showed that a rapid and clear response towards etoposide at a potential of +0.27 V was observed, and the current response increased with increasing concentrations of etoposide ranging from 0.1 μM to 500 μM. There were two linear relationships between the oxidation peak current density and the etoposide concentration in the range of 0.1–20 μM and 20–150 μM. Under lower etoposide concentrations of 0.1–20 μM, the linear regression equation was j (μA cm^−2^) = 0.6818 × C_Eto_ (μM) +2.571 (R^2^ = 0.98), and the sensitivity reached 681.8 μA mM^−1^ cm^−2^. At higher concentrations of 20–150 μM, the linear regression equation was j (μA cm^−2^) = 0.1978 × C_Eto_ (μM) +16.74, and the sensitivity reached 197.8 μA mM^−1^ cm^−2^. The limit of detection (LOD) by this method reached 20 nM. These results indicated that the NPG/GCE constructed in this study had a good etoposide detection performance that may be used in real sample analysis.

A comparison of the NPG/GCE sensor with existing sensors for the measurement of etoposide is presented in Table 1, which shows that the NPG/GCE has a wide detection range and also has the potential to detect etoposide at higher concentrations. Also, the simplicity of the NPG construction method, the high absorbability of its porous structure, the high catalytic activity of gold, and the economic benefits confer advantages to the use of NPG for the detection of etoposide. In addition, etoposide is an antitumor drug, and the NPG/GCE sensor has the ability to detect a number of tumor markers, such as carcinoembryonic antigen and lung-cancer-specific exosomes [34,35,36]. Thus, NPG has the potential to be applied to harmonize the co-testing of therapeutic drugs and physiological indicators.

### 2.5. Anti-Interference of Etoposide Detection

**Figure 5:**
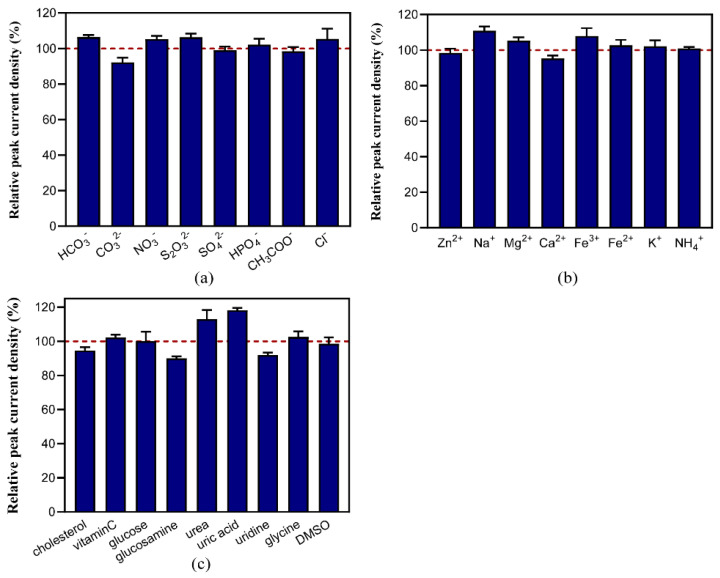
The anti-interference abilities of etoposide detection using the DPV method. The deviation rate of the peak current by the disturbance of (**a**) anions, (**b**) cations, and (**c**) some compounds that exist in biological samples was analyzed. The detection was performed in 50 mM of phosphate buffer (pH 7.4) at a scan rate of 50 mV s^−1^ from −0.4 V to +1.0 V. The figures show the average of three replicate measurements, red dotted line indicates the relative peak current density of 50 μM etoposide with no interfering substances.

Anti-interference ability is also an important index to evaluate the performance of a sensor. We analyzed the common interfering substances in etoposide biosynthesis and analysis for the biological samples. Interfering substances were added at 5 times (uridine 1 time) the concentration of etoposide to the phosphate buffer containing 50 μM of etoposide. The results of DPV responses (Figure 5) showed that the sensor had good anti-interference capabilities to a variety of substances including several common anions and cations, as well as some physiological metabolites and the solvent DMSO. The deviation rate of the peak current density in the presence of these common interferences was usually less than 10%. The impacts of Na^+^ and Fe^3+^ were higher; however, the deviation rate was still less than 16%, and the increase in the peak current may provide a good clue for the improvement in the sensor in preparing the buffer system for etoposide analysis. The addition of urea or uric acid also caused a slight increase in the peak current. An amount of 250 μM of uridine had high impact on the detection; however, when the uridine concentration was less than 50 μM, the sensor showed good anti-interference ability, as shown in Figure 5c. Given that the concentration of uridine in urine is similarly low, it is assumed that uridine will not cause interference in practical assays. 

### 2.6. The Detection of Etoposide in Biological Samples

To explore its application performance in biological samples, we performed spike recovery tests on fermentation broth, fetal bovine serum, and human urine. The detection in fetal bovine serum was performed in 100% serum. As the injection dosage of etoposide in the human body is normally 100 mg m^−2^ per day [44], we chose 5, 10, and 20 μM as the spiked concentrations in serum. The results showed that the recovery rates of spiked etoposide in serum samples were between 96 and 97.7%, with a standard deviation rate lower than 7.91% (Table 2).

The detection in urine was performed with spiked etoposide at 5, 20, and 100 μM. The components of human urine samples are complicated, and some substances in urine can bind nonspecifically to the recognition element and disturb the detection [24]. Our results in the anti-interference test also showed the disturbance of urea and uric acid at high concentrations (Figure 5c). Thus, the urine was diluted in the potassium phosphate buffer 100 times to prevent the matrix effects. At this urine concentration, the etoposide recovery rates at medium and high concentrations were 101.25% and 103.88%, respectively, with standard deviation rates lower than 4.69%. The disturbance of the urine component on the detection of low etoposide concentration was relatively high, with a recovery rate of 84.6% and a standard deviation rate of 6.15%.

In the recovery experiments of etoposide in the fermentation broth, the low, medium, and high concentrations of 10, 50, and 150 μM of etoposide were added to the supernatant of *E. coli* overnight fermentation broth, and the recovery rates were between 91.32% and 107.93%, with a standard deviation rate lower than 6.94%.

Spike recovery tests were also performed in serum and urine using the HPLC method (Table 2). It was found that the existence of biological samples has large impacts on the detection of etoposide under low concentrations, with no reproductible data obtained below 10 μM. This showed that the NPG/GCE sensor had a high LOD, better specificity, and immunity to interference compared to the HPLC method. An extraction process may be required for the detection of low-concentration samples. 

The detection of etoposide in biological samples was always limited by the presence of the interfering substance, and, because of the complexity of the patient’s physical conditions, their blood and urine samples will also show large differences, which also brings certain difficulties to the detection of the biological samples. In our study, the detection in pure urine received severe interference, and the detection of etoposide under low concentrations (below 2 μM) in the three biological samples showed poor reproducibility. This limited its usage in some cases, so the addition of some modifications of the electrode may be helpful.

## 3. Materials and Methods

### 3.1. Chemical and Materials

Etoposide was purchased from Macklin (Macklin, China). All other chemicals were of analytically pure grade. Glassy carbon electrodes (GCEs), saturated calomel electrodes (SCEs), and α-Al_2_O_3_ powder 0.05 μm) were purchased from Shanghai Chenhua Instrument Co., Ltd. (Shanghai, China).

### 3.2. Preparation of NPG/GCEs

The dealloying method for the preparation of NPGs used thin sheets of gold–silver alloys (1:1, *w*/*w*) with a thickness of 100 nm, which were first cut into 7 × 7 mm squares and dealloyed in concentrated nitric acid at 40 °C for 1 h to remove the silver atoms. Nanoporous gold was obtained by washing three times using ultrapure water after etching. GCEs were polished with a 0.05 μm alumina slurry on a piece of chamois leather. Before use, the GCEs were ultrasonically cleaned using ultrapure water and ethanol. After that, nanoporous gold pieces could be covered on the GCEs. An amount of 3 μL of 0.1% Nafion solution was dropped onto the constructed NPG/GCEs. After drying, the NPG/GCEs were activated by cyclic voltammetry (CV) scans in 0.5 mol L^−1^ H_2_SO_4_ solution. The voltage ranged from +0.35 V to +1.55 V at a rate of 50 mV s^−1^. Scans were repeated until a stable cyclic voltammetry curve was observed.

### 3.3. Electrochemical Analysis

The nanoporous films were characterized by scanning electron microscopy (SEM, FEI Quanta 250 FEG, Thermo Fisher Scientific, Waltham, MA, USA) to examine the surface of the NPG/GCE.

Cyclic voltammetry (CV) and differential pulse voltammetry (DPV) were performed using the CHI760E electrochemical workstation (Chenhua, China). Three electrodes were used in the electrochemical measurement. NPG/GCEs worked as the working electrodes, with the Pt electrodes acting as the counter electrodes and the saturated calomel electrodes performing as the reference electrodes.

An electrochemical performance test was executed in 50 mM potassium phosphate buffer electrolyte (pH 7.4) containing 5 mM potassium ferricyanide and 5 mM potassium hexacyanoferrate (II). The CV parameters were set as follows: potential range, −0.8–1.25 V; sweep speed, 50 mV s^−1^; 3 turns. EIS parameters were set as follows: frequency range, 10^−2^–10^4^ Hz; AC voltage, 5 mV s^−1^. CV and DPV detection were performed in 50 mM potassium phosphate buffer (pH 7.4). The DPV parameters were set as follows: voltage range, −0.4–+1.0 V; pulse voltage, 25 mV; step voltage, 5 mV.

### 3.4. Actual Sample Detection

Some substances in the samples can bind nonspecifically on the recognition element to produce a matrix effect, which may affect sensor detection [24]. Thus, the analysis of some biological samples was performed by dilution of the samples in the 50 mM potassium phosphate buffer (pH 7.4) to prevent the matrix effects. The urine samples were taken from a researcher and diluted 100-fold after centrifugation at 12,000× *g* for 10 min. All the spiked recovery tests used the same sample; the fermentation broth was taken from the supernatant of *E. coli* BL21 (DE3) overnight culture in M9 medium followed by centrifugation at 12,000× *g* for 10 min. The fetal bovine serum was purchased from Thermo Fisher Scientific. 

### 3.5. HPLC Analysis

High-performance liquid chromatography (HPLC) analysis was carried out on a Shimadzu LC-20AT HPLC system (Shimadzu, Japan) with an ultraviolet detector for the evaluation of the accuracy of the electrochemical analysis. Etoposide of different concentrations in serum or 1% urine was injected. Akasil C18 column (5 μm, 4.6 mm × 250 mm) was eluted with a gradient of solution A (pure water) and solution B (pure acetonitrile) from 7.5% B to 52.5% B in 15 min, 52.5% B for 5 min, and 100% B for 11 min at a flow rate of 0.8 mL/min. The detection wavelength was 254 nm.

## 4. Conclusions

In this study, we successfully prepared an NPG/GCE using the dealloying method using Au-Ag alloy film and characterized it using SEM, CV, and EIS to confirm the successful preparation of the electrode. Then, we identified the optimum buffer pH for etoposide analysis and obtained the linear relationship between etoposide concentration and peak current density using DPV analysis. The linear regression equation was j (μA cm^−2^) = 0.6818 × C_Eto_ (μM) + 2.571 (R^2^ = 0.98) at etoposide concentrations between 0.1 and 20 μM, with a sensitivity reaching 681.8 μA mM^−1^ cm^−2^; the linear regression equation was j (μA cm^−2^) = 0.1978 × C_Eto_ (μM) + 16.74 at etoposide concentrations between 20 and 150 μM, with a sensitivity reaching 197.8 μA mM^−1^ cm^−2^. The LOD of etoposide using this method reached 20 nM. The reproducibility of the electrode was good. Hence, the electrode was used to detect etoposide in biological samples, and the relative standard deviation in the recovery experiment was reasonable.

## 5. Patents

Chinese Patent (application no. 2023116743639) resulted from the work reported in this manuscript.

## Figures and Tables

**Table 1:** Comparison of NPG/GCE sensors with other sensors.

Electrodes	Linear Range (μM)	LOD (nM)	Sensitivity (μM μA^−1^)	Methods	References
CEZLNCs/CPE	0.04–120	2.7	0.2933	DPV	[37]
CQDs/GCE	0.06–100	17	0.4376	DPV	[38]
Au/Pd@rGO@p(L-Cys)/PGE	0.01–40.0	0.718	1.1319	DPV	[19]
GO/CoFe_2_O_4_/LDH/FTO	0.2–10	1	63.408	DPV	[39]
MWCNT/GCE	0.02–2	5.4		AdSDPV	[40]
Carbon Paste (CPE)	0.25–25	100	0.29436	DPV	[41]
dsDNA/SPCE	0.034–0.68	5		DPV	[42]
GNR/GCE	0.4–150	100	0.1598	CV	[16]
PG	0.705–6.34	156	0.6621	SWV	[43]
NPG/GCE	0.1–150	20	0.4636	DPV	This work

CEZLNCs/CPE: a carbon paste electrode (CPE) modified with a new green synthesized nanocomposite, which uses Cuscuta epithymum extract as a reduction and capping agent for the green synthesis of Zno-Lapis lazuli nanocomposite. CQDs/GCE: carbon quantum dots loaded on the GCE. Au/Pd@rGO@p(L-Cys)/PGE: pencil graphite electrode modified with Au nanocomposite decorated with poly (L-Cysteine). GO/CoFe_2_O_4_/LDH/FTO: ZnAl/layered double-hydroxide-modified cobalt ferrite–graphene oxide nanocomposite electrophoretically deposited (EPD) on the fluorine tin oxide (FTO) substrate. GNR/GCE: graphene nanoribbon nano-catalyst loaded on the GCE. PG: a disposable pen-tip electrode. MWCNT/GCE: a multi-walled carbon nanotube-modified glassy carbon electrode.

**Table 2:** Spiked recovery experiments of the NPG/GCE in the biological samples.

Samples	Spiked Etoposide (μM)	Detected by NPG/GCE (μM)	Recovery Rate (%)	Deviation Rate (%)	Detected by HPLC (μM)
Fetal Bovine Serum	5	4.80 ± 0.38	96.00	7.91	ND
	10	9.77 ± 0.41	97.70	4.20	ND
	20	19.36 ± 0.76	96.80	3.92	18.75 ± 1.12
Human Urine	5	4.23 ± 0.26	84.60	6.15	ND
	20	20.25 ± 0.77	101.25	3.80	25.36 ± 8.62
	100	103.88 ± 4.87	103.88	4.69	87.17 ± 1.03
Fermentation Broth	10	10.50 ± 0.73	105.00	6.90	
	50	45.66 ± 0.64	91.32	1.40	
	150	161.89 ± 1.58	107.93	0.98	

ND: not directly detectable, required extraction prior to detection.

## Data Availability

The data presented in this study are available on request from the corresponding author.

## Supplementary Materials

The following supporting information can be downloaded at https://www.mdpi.com/article/10.3390/molecules29051060/s1. Figure S1: Reproducibility of NPG/GCE sensors.
