# Supplementary material for: Rapid Detection of the Anti-Tumor Drug Etoposide in Biological Samples by Using a Nanoporous-Gold-Based Electrochemical Sensor
Source: Molecules. 2024 Feb 28;29(5):1060. doi: 10.3390/molecules29051060 (PMC10933961; doi:10.3390/molecules29051060)
Supplement: Supplementary file 1 [file molecules-29-01060-s001.zip › molecules-2841891-supplementary.pdf]

Rapid detection of the anti-tumor drug etoposide in biological samples by using a nanoporous gold-based electrochemical sensor

Huiyuan Yu [1], Mengjie Hu [1], Xiaolei Wang [1], Xia Wang [1], Luying Xun [1,2] and Honglei Liu [1,*]

Content:

Figure S1

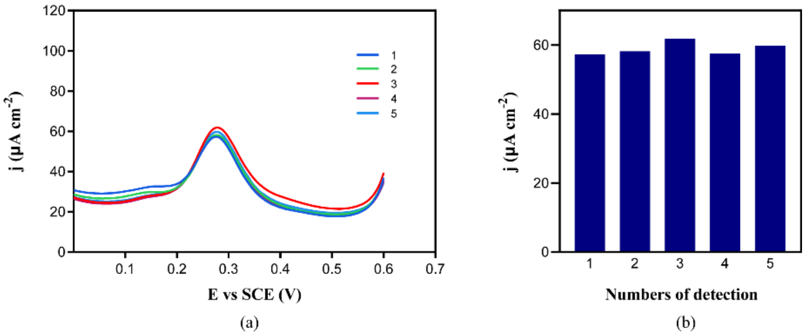

Figure S1 Reproducibility of NPG/GCE sensors. (a) The DPV curves of 100 μM etoposide in 50 mM, pH7.4 phosphate buffer for 5 measurements. (b) The peak current density for the five measurements.
